# Research Progress of Macrophages in Bone Regeneration

**DOI:** 10.1155/2023/1512966

**Published:** 2023-02-07

**Authors:** Dingmei Zhang, Yi Dang, Renli Deng, Yaping Ma, Jing Wang, Jun Ao, Xin Wang

**Affiliations:** ^1^Department of Orthopaedic Surgery, Affiliated Hospital of Zunyi Medical University, Zunyi, Guizhou 563003, China; ^2^Nurse Department, Affiliated Hospital of Zunyi Medical University, Zunyi, Guizhou 563003, China

## Abstract

Bone tissue regeneration plays an increasingly important role in contemporary clinical treatment. The reconstruction of bone defects remains a huge challenge for clinicians. Bone regeneration is regulated by the immune system, in which inflammation is an important regulating factor in bone formation and remodeling. As the main cells involved in inflammation, macrophages play a key role in osteogenesis by polarizing into different phenotypes during different stages of bone regeneration. Considering this, this review mainly summarizes the function of macrophage in bone regeneration based on mesenchymal stem cells (MSCs), osteoblasts, osteoclasts, and vascular cells. In conclusion, anti-inflammatory macrophages (M2) have a greater potentiality to promote bone regeneration than M0 and classically activated proinflammatory macrophages (M1). In the fracture and bone defect models, tissue engineering materials can induce the transition from M1 to M2, alter the bone microenvironment, and promote bone regeneration through interactions with bone-related cells and blood vessels. The review provides a further understanding of macrophage polarization behavior in the evolving field of bone immunology.

## 1. Introduction

Fracture does harm to human health and more than 20 million fractures annually because of osteoporosis, osteosarcoma, osteomalacia, osteomyelitis, and atrophic nonunion [1]. Bone defects are mostly caused by fractures complicated by infection, severe trauma, improper postfracture management, or other comorbidities [2]. Only a quarter of people with fractures have received orthopaedic treatment, and more than half of them have treated with bone grafts for the affected area [3, 4]. Autologous bone grafting is still considered as the “gold standard” for repairing and reconstructing the bone tissue [2]. However, there are also some disadvantages in autologous bone transplantation such as limited number of transplanted tissues and unsatisfactory location of bone donors. The challenge is to regenerate the bone tissue and sequentially restore bone function. Therefore, in-depth study of the bone regeneration process and intervention measures is expected to find an effective way to treat bone defects.

Inflammation is an important factor to regulate bone formation and remodeling, and the research reports on bone regenerative immune regulation have increased rapidly in recent years. Macrophages, as important immune cells involved in inflammation, can be polarized into proinflammatory M1 phenotype or anti-inflammatory M2 phenotype in different microenvironments and express and release different cytokines, thereby regulating bone regeneration at different stages [5]. Pajarinen et al. have shown the different roles of macrophages in intramembranous osteogenesis and intrachondral osteogenesis of fracture healing and normal bone homeostasis [6]. For example, macrophages contribute to woven bone deposition during intramembranous osteogenesis while promoting the formation of cartilage callus in the process of endochondral osteogenesis [7, 8]. In this review, we not only described the regulation of macrophages on bone regeneration from the aspects of macrophages on MSCs, osteoblasts, osteocytes, osteoclasts, and bone blood vessels but also expounds the application of macrophages in the treatment of bone regeneration with biomaterials, which could provide ideas for further research on the mechanism of the effect of macrophages in bone repair as well as the targeted repair and regeneration of bone tissue by regulating macrophages.

## 2. Macrophage Phenotypes

**Figure 1:**
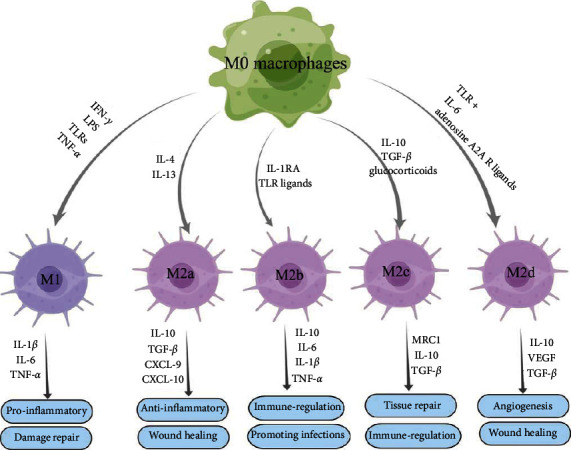
The polarization of M0 macrophages toward M1 and M2 phenotypes and their secretory factors and functions. M0 macrophages can be polarized into M1 or M2 phenotypes under different inducing factors. M2 macrophages are further classified into 4 subsets: M2a, M2b, M2c, and M2d. These cells secrete different cytokines and participate in cellular inflammatory response and tissue injury repair.

Macrophages are innate immune cells that exist in almost all tissues and play a key role in maintaining normal tissue homeostasis due to their heterogeneity and plasticity [9, 10]. Through the action of phagocytosis, cytolysis, and release of inflammatory factors, macrophages clear the exogenous microorganisms to restore the body dynamic equilibrium, and under the action of cytokines, macrophages change their phenotype to enhance their ability to respond to changes in the microenvironment; this process is known as macrophage polarization [11]. Under different microenvironmental conditions, the tissue‐resident macrophages (M0) are pluripotent cells that exhibit a high degree of plasticity, which could display the phenotypic (M1 and M2) and functional differentiation, showing marked heterogeneity [11, 12]. Among them, M1 macrophages can be activated by IFN-*γ*, lipopolysaccharide (LPS) or Toll-like receptor (TLR), and tumor necrosis factor-alpha (TNF-*α*) (Figure 1). M1 macrophages highly express inducible nitric oxide synthase (iNOS), CD80, and CD86 genes, killing and clearing pathogens by producing reactive oxygen intermediates (ROIs), NO, and releasing lysosomal enzymes. The M1 macrophages originated chemokines including CD62L/CD62L ligands, CX3CR1/CX3CL1, CCR2/CCL2, and VEGFR1/VEGF-A proinflammatory cytokines such as IL-1*β*, IL-6, and TNF-*α* also are involved in inflammation and repair of damaged tissues and cells [13–16] (Figure 1). M2 can be induced and activated by IL-4 or IL-13 and secrete transforming growth factor-*β* (TGF-*β*), IL-4, IL-10, and IL-13. IL-10, and other anti-inflammatory cytokines inhibits inflammation and promotes remodeling of the damaged tissue [13, 15, 17] (Figure 1). The immunomodulatory role of macrophages is reflected in their ability to cooperate with other cell types (such as neutrophils) to resist external adverse factors and to secrete different types of cytokines, including IL-1 and IL-6 to activate other immune cells [18–20]. Although macrophages are essential for efficient control and clearance of infections, for example, they also contribute to tissue damage in infection and inflammatory diseases [21, 22]. M2 macrophages could be further classified into 4 subsets: IL-4/13-induced M2a macrophages which express mannose receptor C-type 1 (MRC1) and IL-10 could promote anti-inflammatory response and wound healing; IL-1R agonist (IL-1RA) and TLR ligands-induced M2b macrophages could express IL-10 and major histocompatibility complex class II; IL-10, TGF-*β*, and glucocorticoid-induced M2c macrophages could express MRC1, IL-10, and TGF-*β* to enhance tissue repair. M2d macrophages induced by TLR with IL-6 or adenosine A2A receptor ligands can overexpress vascular endothelial growth factor and inducible nitric oxides synthase (iNOS) or underexpress TNF-*α* and arginine Arginase 1 (Arg1), which play an important role in angiogenesis and wound healing [23, 24]. In damaged tissues, macrophages firstly polarize into M1-phenotype macrophages to produce the proinflammatory response, and it helps the host fight off pathogens by engulfing invading microbes, amplifying the inflammatory response, and recruiting more immune cells. When tissue damage is cleared, macrophages are polarized into M2 phenotypes to form an anti-inflammatory response that repairs damaged tissue by secreting anti-inflammatory factors, recruiting progenitors, and producing growth factors that regulate cell differentiation [25].

## 3. The Regulatory Role of Macrophages in Bone Regeneration

**Figure 2:**
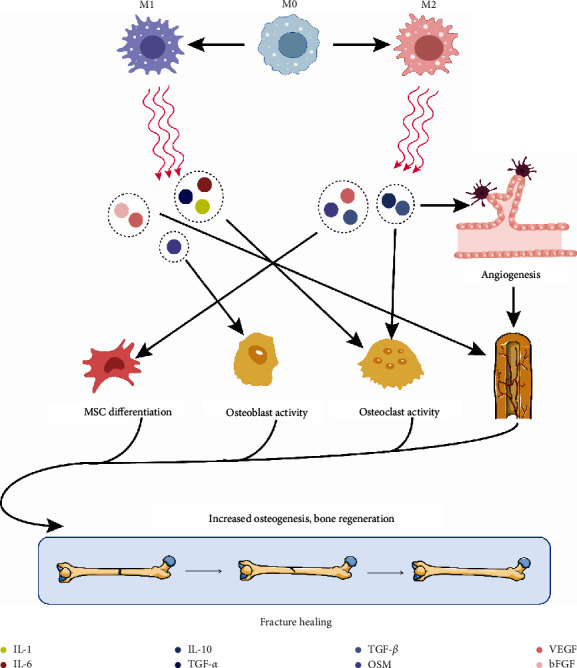
The role of macrophages in the differentiation/function of MSC, osteoblast, and osteoclast. M1 and M2 macrophages secrete the corresponding cytokines to act on MSC, osteoblasts, osteoclasts, and angiogenesis, respectively, so as to participate in bone regeneration during fracture healing.

The function of macrophages within the process of fracture healing has also been confirmed by a number of animal experiments in which macrophage function is knocked out. In the tibial fracture model [26], callus formation and bone deposition are significantly reduced in rats with knockout of macrophage function and are replaced by a large amount of fibrous tissues. In the early stages of fracture, macrophage can be recruited by IL-6 and chemokine receptor 2 (CCR2) released by neutrophil enriched in the fracture region [27] and clearance of primary fibrous tissue and necrotic cells by phagocytosis; they also produce inflammatory mediators and chemokines such as TNF-*α*, IL-1*β*, IL-6, and monocyte chemotactic protein-1 (MCP-1) to recruit fibroblasts, osteoblast precursors, and MSC in the bone marrow, periosteum, and capillary [28]. Deletion of macrophages can reduce the emergence of some proteins associated with bone regeneration such as Sox9, Osterix, COL2, and COL X, as well as decrease the growth factors required for bone regeneration such as TGF-*β*, BMP-2, and IL-6, thereby affecting bone regeneration [29]. Cytokines such as IL-4, IL-10, and IL-13 secreted by M2 macrophages can promote bone formation, and proinflammatory factors such as TNF, IL-1, and IL-6 secreted by M1 macrophages can promote bone resorption [30]. Macrophages can also secrete osteoactive cytokines, matrix metalloproteinases (MMP), and bone morphogenetic proteins to affect bone tissue repair and regeneration (Figure 2).

### 3.1. Macrophages Directly Control Bone Regeneration through Regulating Bone Homeostasis

#### 3.1.1. The Role of Macrophages in MSCs

MSCs are precursor cells of the bone and cartilage that could develop into fibroblast colony-forming cells in vitro and regenerate heterotopic bone tissue in vivo, which are crucial in bone formation. Bone is formed by endochondral and intramembranous ossification. For one thing, MSCs differentiate into chondrocytes during growth plate formation, and chondrocytes are replaced by a new bone during endochondral bone growth. For another, MSCs can also differentiate directly into osteoblasts, which are generated by intramembranous ossification in the absence of a cartilage template [28, 31, 32]. The critical role of macrophages in recruiting and regulating the differentiation of MSCs during bone regeneration has received much attention [6]. Lu et al. [33] studied the effect of the direct coculture system of M0, M1, and M2 mouse bone marrow macrophages on the osteogenic differentiation of bone marrow MSCs, and they found that all macrophage subtypes promoted bone formation. Macrophages participate in the regulation of MSC osteogenic differentiation by secreting different mediators, such as bone morphogenetic protein 2 (BMP2), tumor necrosis factor *α* (*α*), oncostatin M (OSM), and exosomes [34–38].

It is generally believed that M1 macrophages are not conducive to osteogenic differentiation through secreting proinflammatory factors [39]. However, M1 macrophages also promote bone tissue repair [40]. The initial inflammatory response and activation of proinflammatory macrophages may contribute to the recruitment of MSCs, osteoprogenitors, and vascular progenitors to the fracture site. M1 macrophages are involved in the early acute inflammatory phase and clearance of fracture site debris, which promote osteogenic differentiation by recruiting MSCs to the fracture site, inducing the expression of BMP-2, RUNX2, and ALP, and thus are indispensable in fracture repair [38, 41, 42]. Studies by Guihard et al. [37] and Nicolaidou et al. [43] showed that M1 macrophages promote osteoblast-mediated bone formation by producing oncostatin M (OSM), which is a cytokine of the IL-6 family. In addition, M1 macrophages could reduce the secretion of osteoprotegerin (OPG) from MSCs when M1 macrophages were cocultured with MSCs in vitro, thereby reducing their ability to act as RANKL receptors, resulting in the upregulation of osteoclast-related gene expression. Therefore, M1-type macrophages can also indirectly enhance osteoclast activity through MSCs. These studies suggest that M1-type macrophages not only negatively regulate but also positively regulate bone tissue repair. The appearance of M1 macrophages in the early stage of inflammation is conducive to fracture healing, but when their proportion increases or exists for too long, it may be an important factor for delayed bone repair or nonunion, so it may be an effective way that M1 macrophages should be intervened in time.

Growth factors secreted by M2 macrophages support MSCs-mediated bone formation in the late stage of fracture healing, which facilitates the repair and regeneration of the bone tissue by promoting MSCs differentiation into osteoblasts [6]. Intriguingly different from the studies by Guihard et al. [37] and Nicolaidou et al. [43], Fernandes et al. [44] and Loi et al. [45] found that OSM produced from M2 macrophages but not M1 macrophages enhanced the osteogenic differentiation of MSCs. However, the relationship between OSM and M1/M2 macrophages and the interaction between MSCs and macrophages remain to be further explored. Cordova et al. [46] found that the expression of bone synthesis-related metabolic factors such as CCL-2/MCP-1, CCL-5/RANTES, and IGF-1 was upregulated during the polarization of M1 to M2. The study by Zhang et al. [47] also verified this view and pointed out that M2 macrophages can also produce BMP-2 to promote osteogenesis. In the coculture setting of macrophages and bone marrow MSCs, M1 macrophages inhibit MSC-mediated osteogenesis by secreting proinflammatory factors, while M2 macrophages significantly promote osteogenesis by expressing growth factors such as TGF-*β*, vascular endothelial growth factor (VEGF), and IGF-1 [48, 49]. Zhang et al. [47] proved that both M1 and M2 macrophages affect the osteogenic differentiation of MSCs, but they work in different ways: M1 macrophages promoted early and midstage osteogenesis but not matrix mineralization; conversely, M2 macrophages resulted in increased matrix mineralization. Taken together, these studies suggest that M2 macrophages can promote bone formation of MSCs, which may reflect the role of M2 macrophages in the later stages of fracture healing.

#### 3.1.2. The Role of Macrophages in Osteoblasts

The difference from other tissues is that in addition to macrophages from bone marrow, there are some tissue-resident macrophages called osteomacs in the bone. Infiltrating macrophages (M1 and M2) are widely distributed in connective tissues, possess strong deformability and the ability to engulf and kill antigenic foreign bodies such as pathogens, and participate in the process of inflammation in regulating the pathological state of the body [50, 51]. In contrast, most tissue-resident macrophages are long-lived cells derived from a transient hematopoietic wave of erythro-myeloid progenitors (EMPs) present in the yolk sac, which have momentous functions in tissue defense and homeostasis [52]. Osteomacs can enhance the process of bone tissue repair and regeneration, regulating the balance between osteoblasts and osteoclasts [53].

Osteomacs have a positive effect on the proliferation, differentiation, maturation, maintenance, and function of osteoblasts [35, 43, 44, 54–56]. In order to evaluate the functional role of macrophages on osteoblasts, Chang et al. [54] used magnetic sorting technology to remove macrophages (F4/80+) and cultured calvarial osteoblasts, and it was found that depletion of macrophages in cell cultures significantly reduced osteoblast mineralization and gene expression (such as osteocalcin), suggesting that macrophages are not only located in the vicinity of osteoblasts in vivo but also have the ability to support their mineralization function [57]. About 15% of osteoblasts become osteocytes, while 30% of osteoblasts become endosteal cells, and the remaining about 40% to 70% of cells are unexplained; presumably, these cells undergo apoptosis and are subsequently eliminated [58]. Thus, the primary fate of osteoblasts is apoptosis. This process is largely invisible on a histological basis due to the clearance efficiency of macrophages. The efferocytotic effect of macrophages on apoptotic osteoblasts in vitro highlights the process from initial macrophage recognition to phagocytosis of entire osteoblasts [57].

#### 3.1.3. The Role of Macrophages in Osteoclasts

Osteoclasts play a complex role in bone growth and regeneration [59, 60]. Macrophages and osteoclasts share common progenitor cells (myeloid lineage), growth factors (especially macrophage colony-stimulating factor 1, CSF1), and many molecular markers. In the study by Baranowsky et al., macrophage migration is inhibited, thereby restraining the osteoclastogenesis and reducing bone resorption [61]. M2 macrophages promote osteogenic differentiation and inhibit osteoclast differentiation [62]. M1 macrophages can secrete a large number of inflammatory cytokines such as IL-1*α*/*β*, IL-6, and TNF-*α*, upregulate receptor activator for nuclear factor-*κ*B ligand (RANKL), and enhance osteoclastogenesis and bone resorption, resulting in decreased bone mass expression; whereas M2 macrophages reduce osteoclast differentiation by secreting IL-10 and TGF-*β* [63]. In addition, osteomacs could regulate the process of bone remodeling by recruiting osteoclasts to remove necrotic cells in bone remodeling. These studies suggest that future research may regulate osteoclast-mediated bone resorption through macrophages to regulate bone regeneration, and its specific role and mechanism need to be further studied.

### 3.2. The Role of Macrophages in Endochondral Ossification

An important mechanism of fracture healing is endochondral ossification [64]. The process of endochondral bone repair after injury can be divided into three phases: the initial inflammatory phase, the proliferative phase, and the remodeling phase [65]. Macrophages are present at all stages of fracture healing. The correct guidance of macrophages to initiate the healing cascade and the formation of hard and soft callus are both essential for the process of endochondral ossification [5]. A recent study showed that impaired macrophage function had no obvious effects on the early fracture healing but severely delayed endochondral osteogenesis [8]. Macrophage-deficient mice also exhibit less cartilage callus formation and less bone deposition than mice with normal macrophage populations [56], indicating the importance of macrophages for the regenerative process and their necessity throughout the healing process. IL-4 can promote optimal M1/M2 macrophage distribution, resulting in a microenvironment that promotes healing and enhances cartilage ossification [66]. TGF-*β*1 and TGF-*β*3 are key effectors of chondrogenic differentiation. M2 macrophages can secrete TGF-*β*1 and TGF-*β*3 to promote chondrogenic differentiation of MSCs [67]. Also, M1 and M2 macrophages during the hard callus phase contribute to the mineralization of the hypertrophic cartilage by secreting VEGF and TNF-*α* [12, 68, 69]. The extracellular matrix (ECM) of cartilage has an important part not only on cartilage homeostasis but also on endochondral ossification [64]. Decreased M2 macrophages results in loss of ECM remodeling, which delayed endochondral ossification [8, 70]. In addition to the above factors, the prochondrogenic cytokine IL-10 has also been found to be produced by M2-type macrophages and promote tissue regeneration (Figure 2) [69]. In conclusion, the polarization of macrophages to M2 phenotype was induced in the bone healing microenvironment, and some regulatory cytokines were released, which has a positive effect on promoting endochondral ossification. These studies indicated that targeting the macrophages in the treatment of fracture in the future and converting the proinflammatory response to the anti-inflammatory response in time will prevent the delay of bone regeneration and thereby reduce the recovery time of patients.

### 3.3. Macrophages Regulate Angiogenesis during Bone Repair

Bone is a highly vascularized tissue. Bone blood vessels play an important role in bone growth and development, bone defect repair, and bone metabolism balance by providing bone tissue with oxygen, nutrients, hormones, cytokines, and other substances. Osteogenesis and angiogenesis are closely linked in the process of bone formation and repair [71, 72]. Blood vessels not only deliver oxygen and nutrients to the developing bone but also play an active role in bone formation and remodeling by mediating the interaction of osteoblasts, osteocytes, osteoclasts, and vascular cells at various levels [73, 74]. The decline of the angiogenesis ability of the bone microenvironment can weaken the ability of bone formation and lead to bone loss, which is also one of the important reasons for senile osteoporosis [75, 76]. Local blood circulation is destroyed after bone injury, resulting in anoxia and acute necrosis of adjacent bone marrow, resulting in impaired bone formation [77, 78]. Therefore, it is believed that blood vessels are the necessary condition for bone regeneration. Without angiogenesis, there is no bone regeneration. After the fracture, a hematoma firstly forms around the site, followed by callus formation and remodeling. Thus, angiogenesis at the site of bone injury may play a crucial role in bone healing [79, 80].

Angiogenesis includes the activation and proliferation of endothelial cells, the degradation of original vascular basement membrane and extracellular matrix, and the migration and anastomosis of damaged endothelial cells. Cytokines such as IL-8, IL-10, TNF-*α*, and IL-1*β* could promote angiogenesis. TNF-*α* secreted by tumor macrophages can promote the production of angiogenic factors such as IL-8, basic fibroblast growth factor (bFGF), and VEGF [81]. The study of White et al. [82] found that macrophages under hypoxia have increased expression of more than 30 angiogenic genes and produced cytokines such as VEGF, bFGF, IL-8, angiopoietin, and COX-2. About 15 cytokines are currently known to activate endothelial cells division and migration and cause angiogenesis. Zeisberger et al. used chlorophosphate liposomes to specifically clear macrophages and significantly inhibit angiogenesis [83]. Studies have reported [84] that M1 macrophages can secrete a large amount of VEGF and bFGF, thereby recruiting endothelial cells to form vascular sprouts which contribute to the formation of new blood vessels; M2-type macrophages mainly produce transforming growth factor *β* (TGF-*β*) and IL-10, restrain the activity of cytokines which promote the development of inflammation and the release of scavenger receptors, and recruit various anti-inflammatory cells to the area of tissue regeneration to produce a large number of proinflammatory cells to facilitate angiogenesis. Given the close relationship between angiogenesis and bone regeneration, these studies also reflect the role of macrophages in bone regeneration. In addition, M2 macrophages can regulate bone regeneration by promoting angiogenesis, participating in OSM regulation, differentiating into osteoclasts, promoting the osteogenic differentiation of MSCs, and phagocytosing apoptotic MSCs [85]. In bone healing, inflammation, angiogenesis, and new bone regeneration are closely related, and the correct sequence of inflammatory and anti-inflammatory signals is critical for normal bone healing [86].

In the field of regenerative medicine, vascularization has become the fourth element in addition to the three elements of tissue engineering such as scaffold materials, seed cells, and growth factors. Given the critical role of macrophages in promoting angiogenesis, strategies to use these cells to guide the vascularization of scaffolds would be highly advantageous. Therefore, the researchers used biomaterial properties to manipulate macrophages to regulate inflammatory responses and subsequent angiogenesis [87–89]. Piller et al. used bone scaffolds to regulate the sequential polarization of macrophages M1 and M2 to regulate angiogenesis and enhance bone healing [90]. Researchers use the scaffold material to regulate the ratio of M1 and M2 macrophages to promote blood vessel formation [91]. In summary, M1 and M2 macrophages are closely related to regeneration and vascularization. They coordinate with each other that are regularly and quantitatively activated in the inflammatory area of the damaged site and are always in a dynamic state during the occurrence and resolution of inflammation. They can produce a variety of angiogenic growth factors such as CEGF, bFGF, and PDGF and, at the same time, degrade and phagocytose the scaffold material in a timely manner, which will provide sufficient space for bone tissue regeneration.

### 3.4. The Effect of Aging on Macrophage Phenotypic and Bone Repair

As previously described (see Section of 3.1), macrophages are heterogeneously differentiated immune cells (M1 and M2-like macrophages). However, the macrophage phenotype changes with aging [92]. Mahbub et al. reported that aging results in a decrease in both M1 and M2 macrophages [93], while other investigators reported that aging leads to the increase of M2 macrophages in the retina, spleen, lymph nodes, and bone marrow [94, 95]. In a recent study, the researchers found that bone marrow macrophages from aged mice had more activated M1-like macrophages and fewer M2-like macrophages than those from young mice [96]. The reason why these two finding contradicting may be due to the different ages of the animals used and the complex nature of the aging process. The authors also demonstrated that senescent macrophages accumulated in the bone marrow secrete grancalcin (Gca) to promote skeletal aging [96]. Furthermore, it is reported that macrophages from young animals secrete rejuvenating factors, including low-density lipoprotein receptor-related protein 1 (Lrp1), which promotes fracture healing, whereas transplantation of macrophages from older mice delays fracture healing [97]. There are several researchers who observed that an increased M1/M2 macrophages ratio in the aged animals contributed to reducing the bone healing capacity [98–100]. Therefore, slow fracture healing in aging bone may be due to an increase in proinflammatory macrophages, but this remains to be investigated due to the challenges of aging research.

## 4. Application of Macrophages Combined with Biomaterials in Bone Regeneration

The increasing need about large bone grafts with good quantity (volume), quality (bone structure), mechanical properties, and biocompatibility occurs clinically. Notably, continuous development and improvement of tissue engineering technology make it possible that bone tissue regeneration is expected to become a highly promising clinical treatment. Further exploration of the mechanism of tissue engineering about bone will contribute to the success of bone regeneration. Harnessing biomaterials-mediated immunomodulatory strategies for regenerating bone defect has been extensively studied [101]. Macrophages play an important role in immune responses to biological materials and scaffold materials interact with macrophages. On the one hand, scaffold materials can regulate the phenotype of macrophages through their physicochemical properties, so that they can secrete appropriate cytokines and angiogenic growth factors at different stages to guide the recruitment, proliferation, and differentiation of vascular endothelial cells and other cells to promote angiogenesis. On the other hand, macrophages can also release anti-inflammatory cytokines and MMPs, participate in the degradation of scaffold materials, and phagocytose them, providing space for MSCs and new blood vessel sprouts to grow to the center of the scaffold [102]. Types of M2 macrophage responses are beneficial to mediating scaffold tissue repair [103]. Materials with high surface hydrophilicity can promote the polarization of macrophages to the M2 phenotype and can promote the differentiation of MSCs aggregated on the surface of the materials to osteoblasts, thus promoting bone repair [104]. At the same time, the nanomaterial structure titanium surface and its surface titanium-mediated biological activity can also significantly increase the aggregation of M2 macrophages, upregulate alkaline phosphatase activity, and increase bone mineralization [105]. Li et al. reported that the PGO-PHA-AG scaffold promoted the healing of periodontal bone in the microenvironment of periodontal inflammation in diabetic patients by inhibiting M1 macrophage polarization and activating M2 macrophages to secrete osteogenesis-related cytokines [106]. The material scaffold designed by Spiller et al. released IL-4 after IFN-*γ*, resulting in continuous polarization of macrophages toward M1 and M2 phenotypes [90]. The sequential polarization system thus enhances vascularization of subcutaneously implanted bone scaffolds in mice [90]. We present the biomaterials and drugs that modulate macrophage polarization to promote osteogenesis in Tables 1 and 2, respectively, based on the search keywords “macrophage” and “osteogenesis” and “materials or drugs.”

Depletion of macrophages leads to the inhibition of osteoinduction by the bone graft material [59], which further demonstrates their role in guiding repair-related osteogenesis. Therefore, macrophages are an attractive therapeutic target for promoting bone regeneration. The cellular components in natural materials [118], the degradability of materials [119–121], the pore size of scaffold material [122, 123], and the surface chemical modification of scaffolding materials [87, 124], all affect the phenotypic transformation of macrophages. In-depth research on these mechanisms will provide a basis for improving the performance of scaffold materials and selecting appropriate MSCs and cytokines, further enhancing the ability to control the phenotypic transformation of macrophages, promoting angiogenesis in tissue engineering, and improving the regeneration efficiency of surrounding tissues. Considering the involvement of macrophages in innate immunity, especially foreign body response, careful consideration and targeted manipulation of the macrophage function are also required when developing biomaterial approaches for tissue repair.

## 5. Summary and Prospects

Bone is a highly mineralized and vascularized tissue, and various cells are involved in the complex process of its regeneration. As the core of coordinating this complex dynamic process, macrophages are important for maintaining bone homeostasis and promoting bone repair. In short, unactivated macrophages contribute to bone formation which may represent a physiological role for intratissue macrophages in maintaining normal bone homeostasis, while activated macrophages can promote osteogenic differentiation of bone marrow MSCs by increasing MSCs proliferation, ALP activity, and expression of osteocalcin and osteopontin, and these effects are partially dependent on BMP-2 [34, 35]. It will be conducive to the formation of a favorable osteogenic immune microenvironment by regulating the timely transition of macrophages from M1 to M2, thereby further promoting osteogenesis [125]. To further elucidate the function of macrophages in bone growth, different cell synthesis can be studied in vitro in the models of human bone regeneration. For instance, macrophages could be cocultured with stem cells which are mixed in calcium phosphate scaffolds to promote bone growth. The ability of some biochemical signaling molecules which secreted by osteocytes to repair bone defects can be studied in an in vitro model of human bone regeneration. These trials are helpful to illuminate the regulatory role of macrophages on bone regeneration in the process of bone merisis and renovation, and the achievements could be used for clinical application of bone defect healing. With further research on the mechanism of macrophage bone repair, new strategies are designed to more accurately regulate the polarization nodes and functions of macrophages to establish appropriate host-scaffold interaction for optimizing the regenerative and repair process, which may be an important new strategy to achieve targeted repair and regeneration of the bone tissue.

## 6. Disclosure

Dingmei Zhang and Yi Dang are considered the co-first authors. An earlier version of this paper has been published as a preprint in the following link: https://papers.ssrn.com/sol3/papers.cfm?abstract_id=4116172.

## Figures and Tables

**Table 1:** Biomaterials modulate macrophage polarization for bone tissue regeneration.

Materials	Functions	Ref
Bone mimetic nano hydroxyapatite particles (BMnP)	Polarizing macrophages toward an M2 phenotype, activating the transcription factor cMaf, and specifically enhancing production of the anti-inflammatory cytokine IL-10 to enhance bone formation	Mokarram and Bellamkonda [107]
Chitosan/agarose/nanohydroxyapatite bone scaffold	Inducing M2 macrophage polarization and effecting the osteogenic differentiation	Kazimierczak et al. [108]
Mesoporous silica and Fe_3_O_4_ composite-targeted nanoparticles loaded with BCL (BCL@MMSNPs-SS-CD-NW)	Inducing macrophage recruitment, polarizing them toward the M2 phenotype, and inducing mesenchymal stem cells toward osteoblastic differentiation	Zhou et al. [109]
Gastrodin-PU/n-HA scaffold	Inducing macrophage polarization toward the M2 phenotype, upregulating the proregenerative cytokines, osteogenesis-related factors, and angiogenesis-related factors	Li et al. [110]
*β*-tricalcium phosphate (*β*-TCP)	Enhancing osteogenic differentiation of BMSCs by inducing macrophage polarization and by regulating the wnt signaling pathway	Zheng et al. [111]
MSN@IL-4	Controlling the sustaining release of IL-4, inducing M2 polarization and proliferative cytokine of macrophagocyte and following inhibiting the apoptosis and NF-*κ*B pathway-associated inflammation of osteoblast	Shi et al. [112]

**Table 2:** Drug modulates macrophage polarization for bone tissue regeneration.

Drug	Functions	Ref
Irisin	Increasing the cell viability of macrophages, differentiating M0 and M1 macrophages into M2 phenotype, and enhancing osteogenesis and mineralization	Mokarram and Bellamkonda [113]

Naringenin	Promoting the transformation of macrophage M2 and significantly inhibiting M1 polarization, which balances bone formation and resorption by regulating macrophage polarization and cytokine secretion	Zhou et al. [114]

Trehalose	Enhancing the expression of M2 macrophage markers (Arg-1 and IL-10) and reducing the polarization of M1 macrophages by decreasing the expression of IL-6 can promote the delayed healing of bone in rats by downregulating proinflammatory mediators and enhancing M2 polarization	Xu et al. [115]

Harmine	Stimulating the macrophages from M1 phenotype to M2 phenotype, thereby increasing anti-inflammatory and bone-related cytokine levels	Wang et al. [116]

Frugoside	Inhibiting M1 polarization of macrophages, thereby reducing the secretion of IL-6 and TNF-*α*, thereby delaying cartilage and extracellular matrix degradation and chondrocyte hypertrophy	Wang et al. [117]
